# DW-MRI of the breast: a pictorial review

**DOI:** 10.1186/s13244-019-0745-3

**Published:** 2019-06-03

**Authors:** Irmak Durur-Subasi

**Affiliations:** University of Health Sciences, Diskapi Yildirim Beyazit Training and Research Hospital, Clinic of Radiology, Ankara, Turkey

**Keywords:** Breast, Diffusion-weighted imaging, Magnetic resonance imaging

## Abstract

In the current era of breast imaging, magnetic resonance imaging (MRI) has an important role. To get its specificity better, some supporting or cooperative tools might be needed. The search for new methods continues and non-contrast MRI trials are seen. With the shorter and easier acquisition, no need for contrast material, diffusion-weighted (DW)-MRI could be the best collaborator. This pictorial review aims to give an overview of the DW-MRI of the breast by means of a set of specially selected cases.

## Key points

- DW-MRI settles down its own tissue contrast without need for contrast injection.
- Diffusion features, relaxation times, and proton concentration determine the contrast on DW-MRI.
- For screening, the appearances of the lesions on DW-MRI have to be known.

## Introduction

Diffusion-weighted magnetic resonance imaging (DW- MRI) is a non-contrast procedure, measuring the motion of water particles in vivo and analyzing microscopic tissue structure: cellularity, membrane integrity, viscosity, fibers, tubules, organelles, and macromolecules [1]. During the acquisition, motion-sensitizing gradients are used. *b* value is the real diffusion weighting or sensitization (s/mm^2^). So DW-MRI settles down its own tissue contrast without the need for contrast injection. Apparent diffusion coefficient (ADC) is the typical extent a water molecule of a concerned tissue occupies as square mm per second. This is the quantification of DW-MRI and means the diffusion in biologic tissues is actually not unrestricted and is controlled by complex appliances.

DW-MRI is nearly always integrated into dynamic contrast-enhanced (DCE)-MRI protocols. The common clinical indications for dynamic contrast-enhanced magnetic resonance imaging (DCE-MRI) are screening of women at high-risk, assessment of the extent of disease for cancer, evaluation of unclear findings on conventional methods or physical examination, and the estimation of the response to neoadjuvant chemotherapy [2]. DCE-MRI provides great anatomic-morphologic detail and kinetic information about the lesions. Although it allows high sensitivity (approaching 100%), it just delivers a moderate specificity (21–100%) for breast cancer detection. Because imaging characteristics demonstrate some overlap for benign and malignant tumors and wide deviations of the positive predictive value (24–89%) have been reported [2–4].

In this clinical setting, breast DW-MRI has been reported to diminish false-positive consequences, excessive interventions, and improve the positive predictive value [2, 5, 6]. It has shown to be potential to increase the accuracy for the lesion characterization on DCE-MRI with multiple studies [7]. However, it has not yet been included in BI-RADS lexicon.

**Fig. 1:**
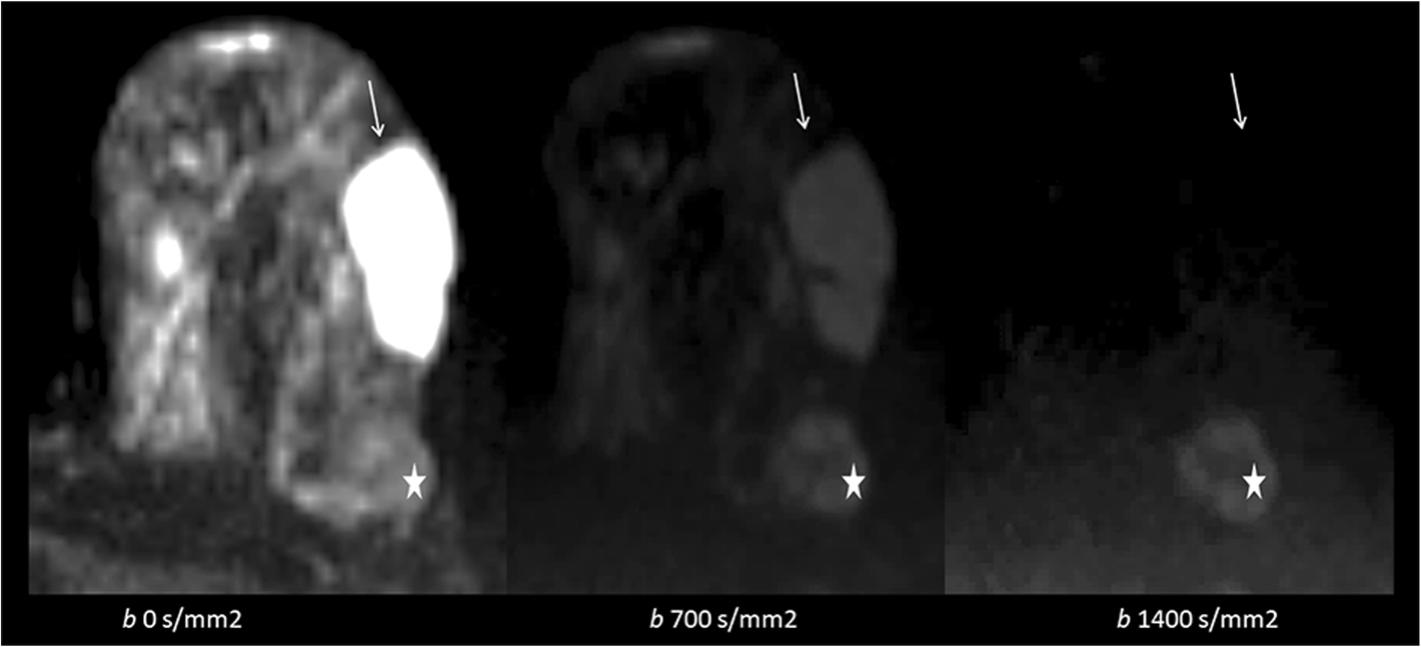
Simple cyst (arrow) and malignant lesion (star) on the left breast. With the increasing *b* value (0, 700, 1400 s/mm^2^), cyst loses its intensity and malignant lesion shows intensity increase. With decreasing T2 effect and increasing diffusion weighting, the diffusion-restricted area becomes more evident (star)

**Fig. 2:**
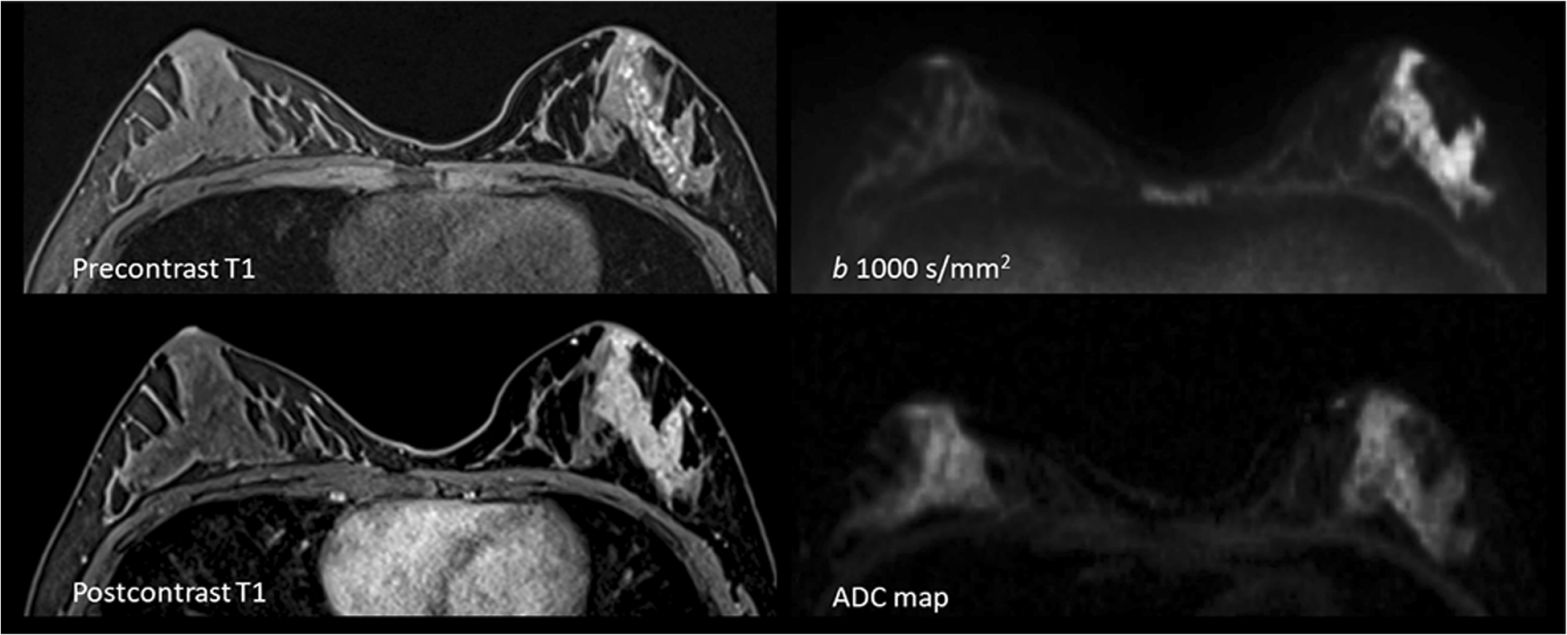
A 23-year-old patient with invasive ductal carcinoma. Hemorrhagic ductal content is seen at the lateral part of the left breast on precontrast T1. There is a segmental enhancement pattern on postcontrast T1. Diffusion restriction is detected on DW-MRI and ADC map. Transverse plane provides a synchronized evaluation

In general, on DW-MRI, malignant lesions show diffusion restriction. Diffusion restriction is appreciated as bright signal intensity (SI) on DW-MRI and having decreased apparent diffusion coefficient (ADC) values. This feature is mostly associated with high cell density and limited extracellular planetary [5]. On the other hand, saw contrast on DW-MRI is determined by not only diffusion features but also relaxation times and proton concentration. The best-known example of this problem is the T2 shine through effect. On DW-MRI with an increasing *b* value, T2 effects will decrease (Fig. 1). T2-effects can be mathematically removed from DW-MRI to create a pure parametric image. This set of data is known as the ADC map. For DW-MRI, any planes can be used. It is recommended to choose the same plane as used for DCE imaging for synchronized evaluation (Fig. 2).

The *b* value provides data on the level of the diffusion weighting and is proportional to the gradient strength and diffusion time. Certain *b* values should be used to standardize ADC thresholds. For lesion detection, high *b* values (800–1500 s/mm^2^) are preferred to create a respectable distinction between the lesion and the neighboring soft tissues [4]. With the aim of benign and malignant discrimination, selection of *b* value is less critical. Performing DW-MRI with more than two *b* values provides a more accurate sampling of the signal drop. However, the breast studies on the use of *b* values more than two have not been shown superiority, therefore two-*b* value DW-MRI is still used as the standard [8]. As the first *b* value, it is also possible to select a *b* value (*b* ≥ 50 s/mm^2^) other than zero to avoid perfusion and flow effects. The diagnostic advantage of such a method for the breast is unclear [9].

Diffusion-weighted imaging of the breast is technically challenging because of the breast being off center within the bore and having intrinsic susceptibility artifacts. However, many technical improvements can be used: proper *b* value choice, adequate signal-to-noise ratio, satisfactory fat suppression, and artifact reduction tools [6].

## What are the clinical applications?

The potential clinical applications are lesion diagnosis and characterization, evaluation of extent of disease, monitoring and predicting treatment response, estimation of prognosis, and biopsy guidance.

### Lesion diagnosis and characterization

ADC value of free water molecules at 37 °C is 3.00 × 10^−3^ mm^2^/s. However, no certain ADC threshold for the benign-malignant discrimination has been determined. Because reported ADC values for breast carcinoma show great variations and DW-MRI protocols are different among the studies or institutions. For qualitative evaluation without an ADC threshold, the lesion is compared to normal fibroglandular tissues on both DW-MRI and ADC map. If the lesion is brighter on DW-MRI and darker on ADC than normal fibroglandular tissue, then it will be classified as diffusion-restricting area. On the other hand, a quantitative evaluation requires ADC measurement.

#### ADC measurement, limitations, and potential solutions

It is recommended to measure the solid portions excluding normal tissue, necrosis, or fat.

The selection of the most appropriate ADC threshold depends on the expectations from the DW-MRI. For breast cancer screening, the threshold must be selected high. However, in order to increase the specificity of dynamic breast MRI, a lower threshold is preferred [6]. The recommended threshold ADC values for benign-malign distinction vary between 0.90 and 1.76 × 10^−3^ mm^2^/s [10, 11]. In general, benign lesions show a higher mean of ADC than malignant lesions, but there is a significant overlap with malignant lesions [6].

**Fig. 3:**
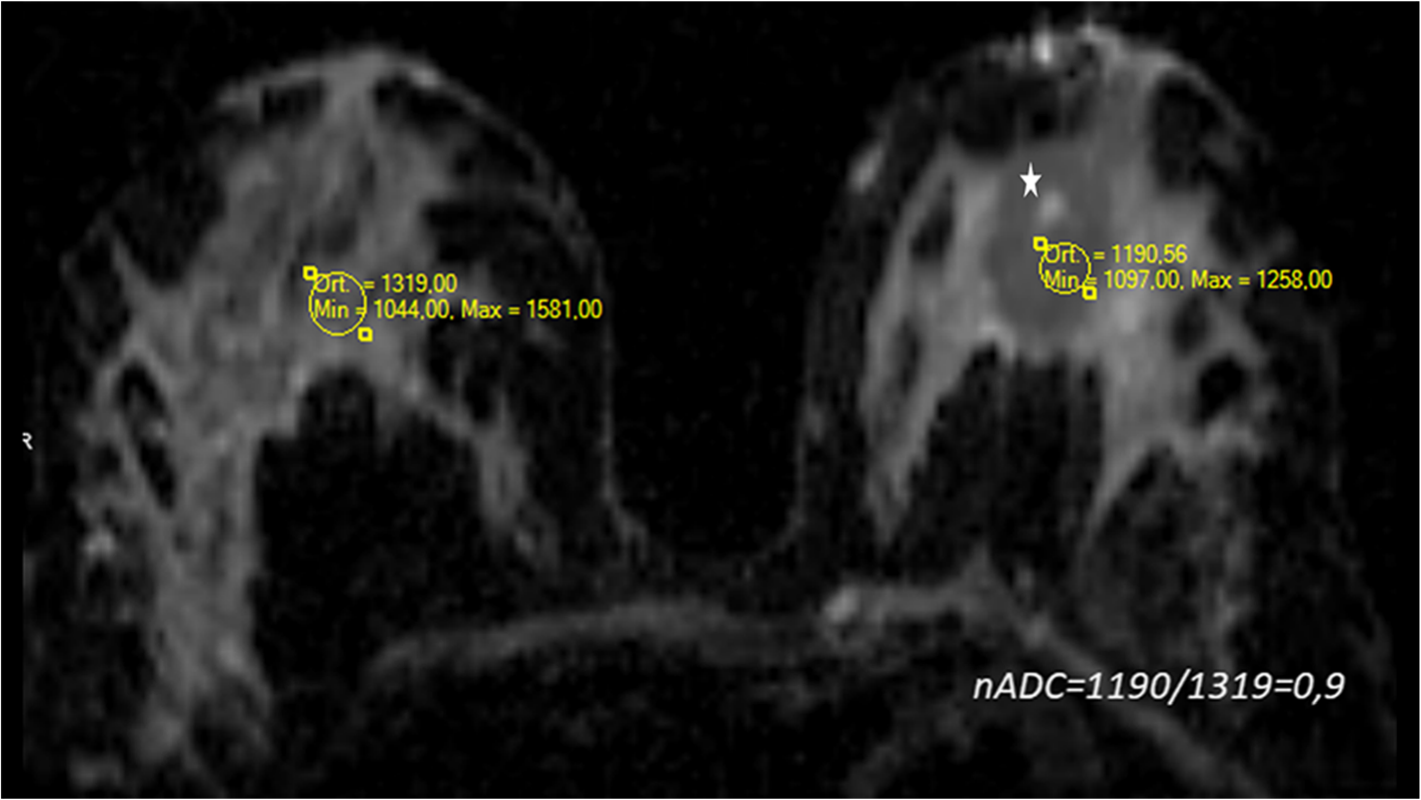
Phyllodes tumor of the breast (star). ADC normalization is seen. nADC shows a little restriction and it is higher than the recommended threshold of 0.7

As mentioned before ADC values for breast carcinoma show great variations. So ADC normalization would be a solution. It is calculated as the ratio of the ADC value of the lesion to that of the normal parenchyma. It was reported that the ADC similarity of benign and malignant lesions is decreased by ADC normalization and subsequently, the accuracy of DW-MRI is improved, variations from individual breast characteristics and technical factors could be reduced. An optimal normalized ADC (nADC) cut-off of 0.7 has been reported to have high sensitivity and specificity rates (Fig. 3). However, further investigations are needed and nADC is limited in case of widespread disease and profuse fat tissue [12].

**Fig. 4:**
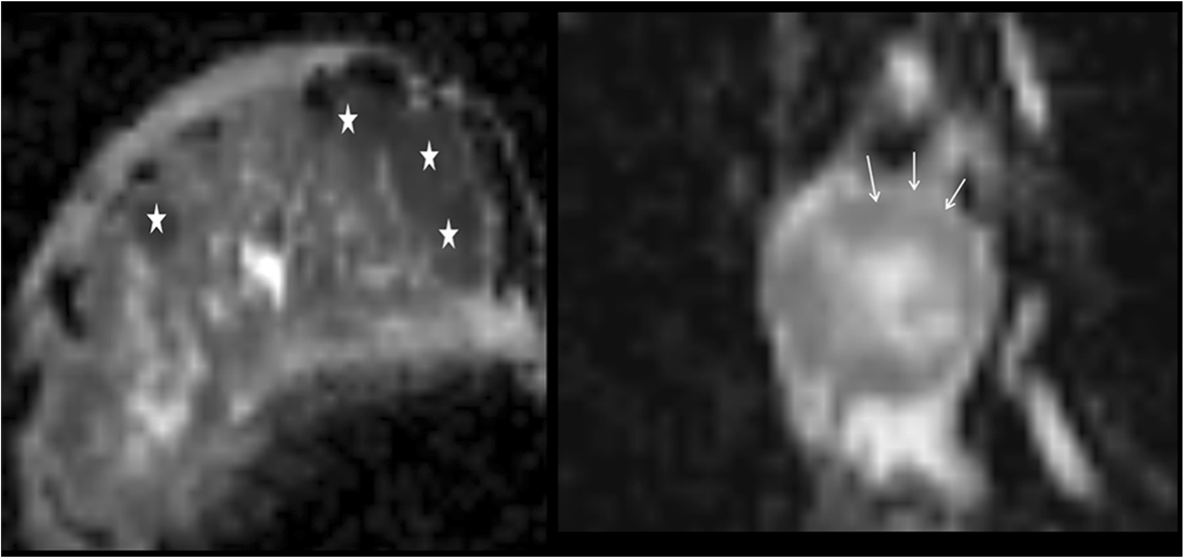
ADC map of two different patients. Necrosis of the abscess (stars) and the wall of the carcinoma (arrows) show diffusion restriction much more. The patient with abscess also has widespread mastitis. Perilesional oedema is seen just around the breast carcinoma

Another point is to take the necrotic portion into consideration when measuring ADC. Necrosis/wall ADC ratio (NWADCr) is the ratio of the ADC values of the necrosis to the wall. It was shown that NWADCr is statistically different for benign and malignant necrotic breast lesions [1]. Wall of carcinoma and necrosis of an abscess will demonstrate more diffusion restriction because of first one having high cellularity, the large size of nucleus, intracellular macromolecules, the limited size of the extracellular matrix, and the second one showing high viscosity and cellular debris. In general, NWADCr will be below 1 for abscess and higher than 1 for carcinoma (Fig. 4).

#### What are the false positives of breast DW-MRI?

**Fig. 5:**
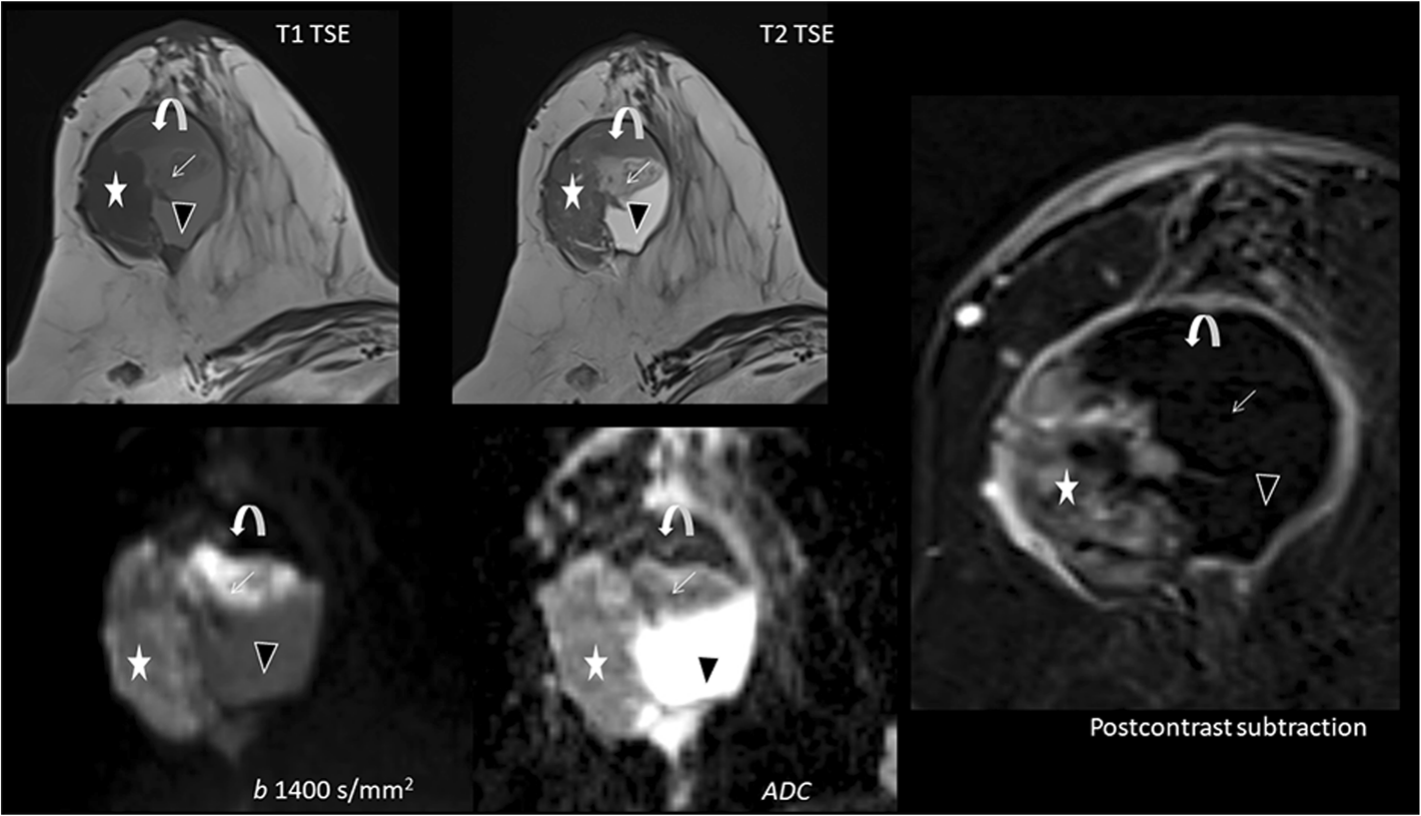
Papillary tumor of the breast. A well-bordered complex cystic mass with the solid portion (star) restricting diffusion. The lesion shows three different appearances of hemorrhage. Appearances of hemorrhagic contents of the tumor depend on several factors. The content located at dependent part (curved arrow) is T1 and T2 hyperintense, and may be superimposed by the susceptibility artifacts due to degradation products and being very hypointense on both DW-MRI and ADC map. The hemorrhagic contents located at the central part of the lesion (arrow) restricts diffusion because bright on DW-MRI and dark on ADC map. This can be due to intact red blood cell membranes with intracellular oxyhemoglobin, deoxyhemoglobin, or methemoglobin and clot retraction. Lastly, the content marked black arrowhead shows no restriction can be explained by disintegration products of the bleeding and more chronic stages

**Fig. 6:**
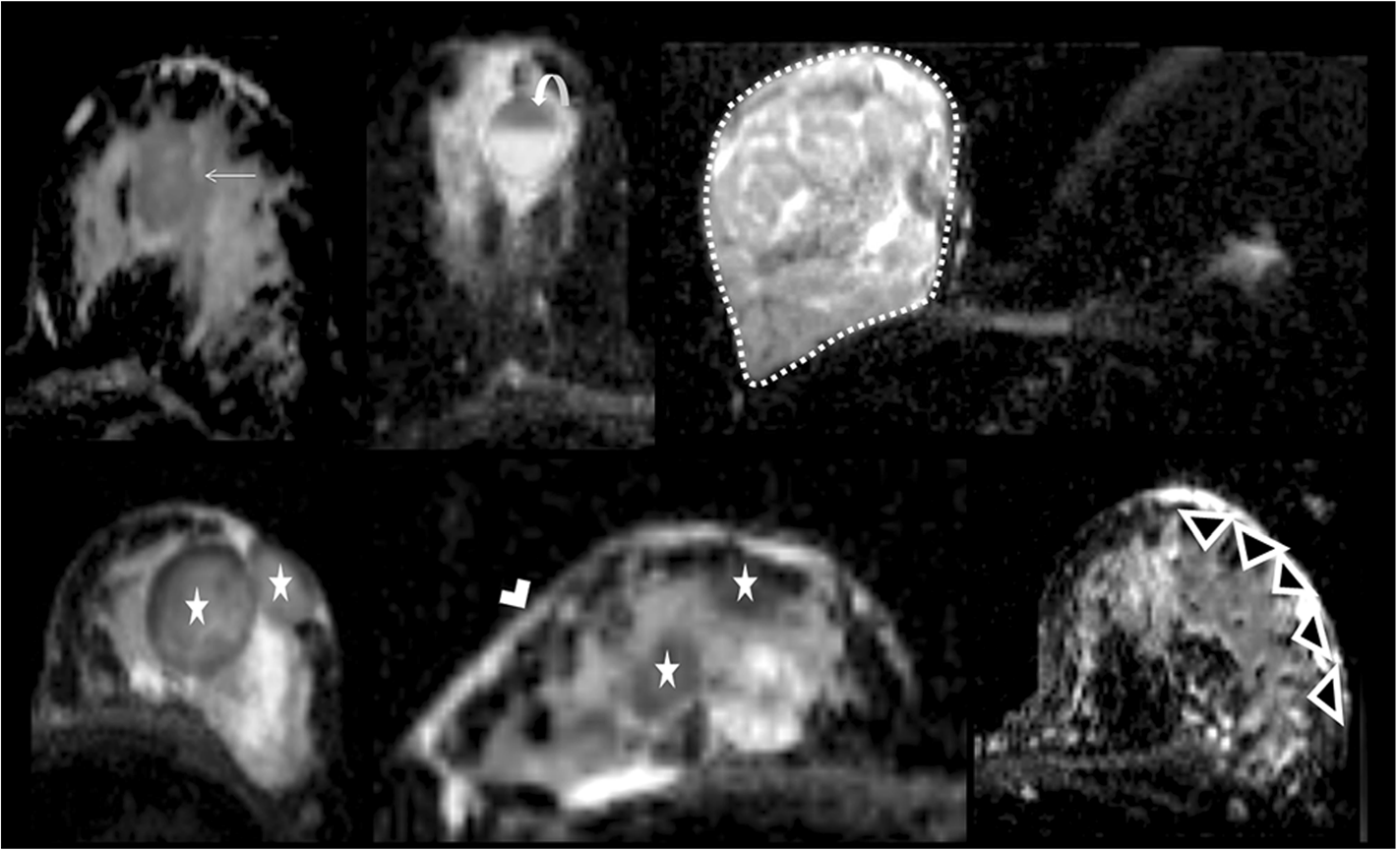
Six different false positive lesions on ADC maps. A highly cellular fibroadenoma is seen as a heterogeneous, focal dark area (arrow). Cyst with thick content (curved arrow) shows fluid-fluid level with dependent portion being thicker. A juvenile fibroadenoma with high cellularity (surrounded by a dashed line, pay attention to the small and normal sized left breast) demonstrates different diffusion features most of which with restriction. Breast abscesses (stars) are restricting diffusion. Note the thickened breast skin marked by white arrowhead. Mastitis (black arrowheads) can show diffusion restriction due to inflammatory cell migration and vascular fibroblastic proliferation

**Fig. 7:**
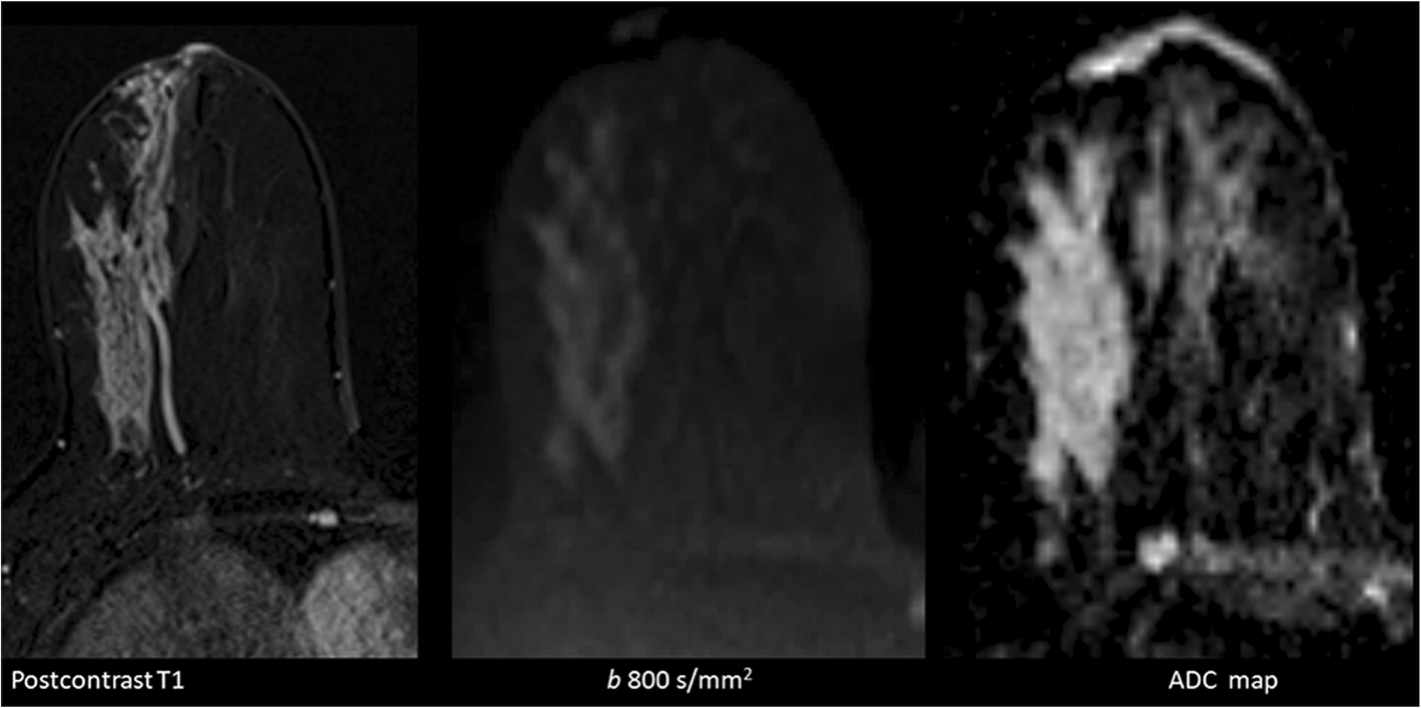
Segmental enhancement and corresponding increased diffusion due to DCIS. The interpretation of DCIS at DW-MRI remains controversial. Depending on the cellularity, diffusion features will change

Hematomas having intracellular oxyhemoglobin, deoxyhemoglobin, or methemoglobin, fibroepithelial lesions with high cellularity, breast abscess, mastitis, cyst with thick content, intramammary lymph nodes, ductal carcinoma in situ (DCIS), intraductal papilloma, and atypical ductal hyperplasia may show diffusion restriction as well (Figs. 5, 6, and 7) [5].

#### What are the false negatives of breast DW-MRI?

**Fig. 8:**
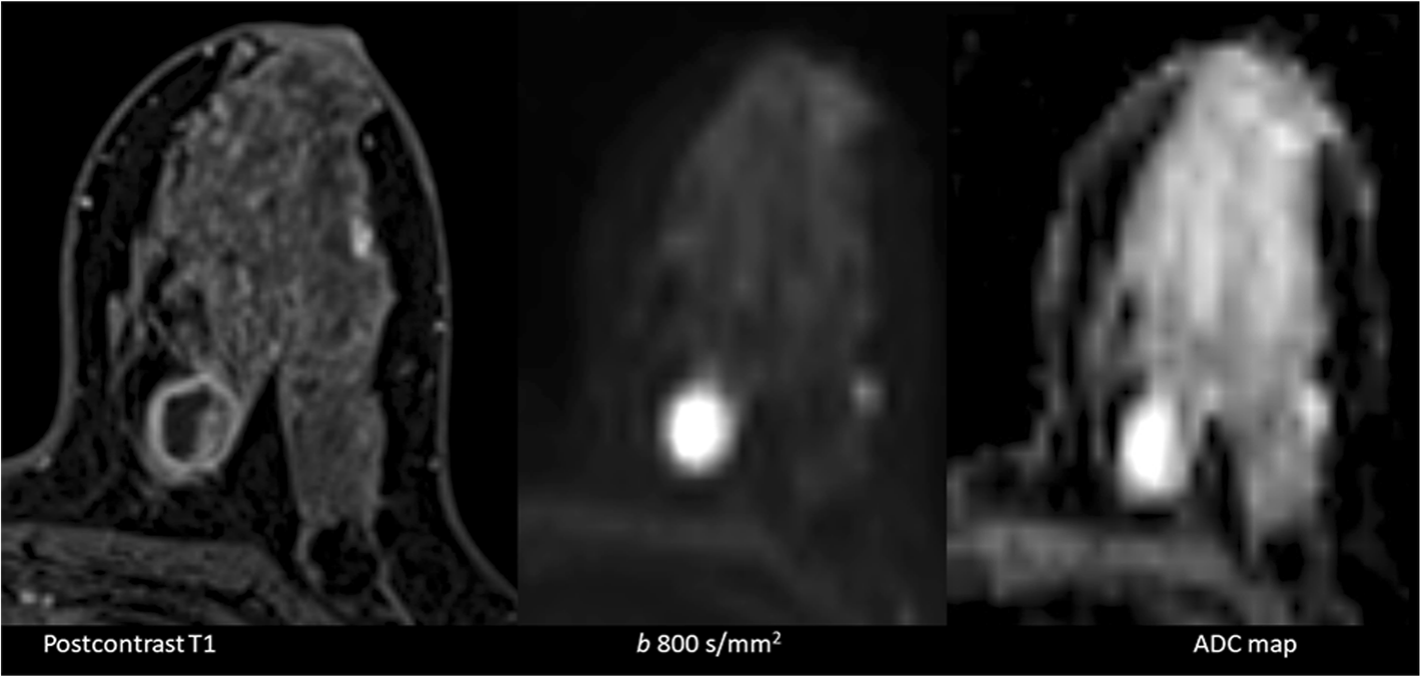
A mucinous carcinoma lesion is seen as a complex cystic lesion and shows no diffusion restriction

Mucinous carcinoma is malignant; however, it will not restrict diffusion due to low cellularity and mucin content (Fig. 8). Additionally, depending on the size of the lesion and field of view and location, some lesions may not be visualized on DW-MRI. It is therefore suggested to combine DW-MRI with other sequences. Additionally, some procedural challenges can affect the quality and some lesions cannot be seen.

### Extent of disease

For multifocal-multicentric breast carcinoma, additional use of DW-MRI in pre-operative setting can benefit to elude unnecessary biopsies by upward specificity. However, according to that study to evade missing cancers, clinicians should closely follow up the lesions of the same quadrant or larger than 1 cm [13].

**Fig. 9:**
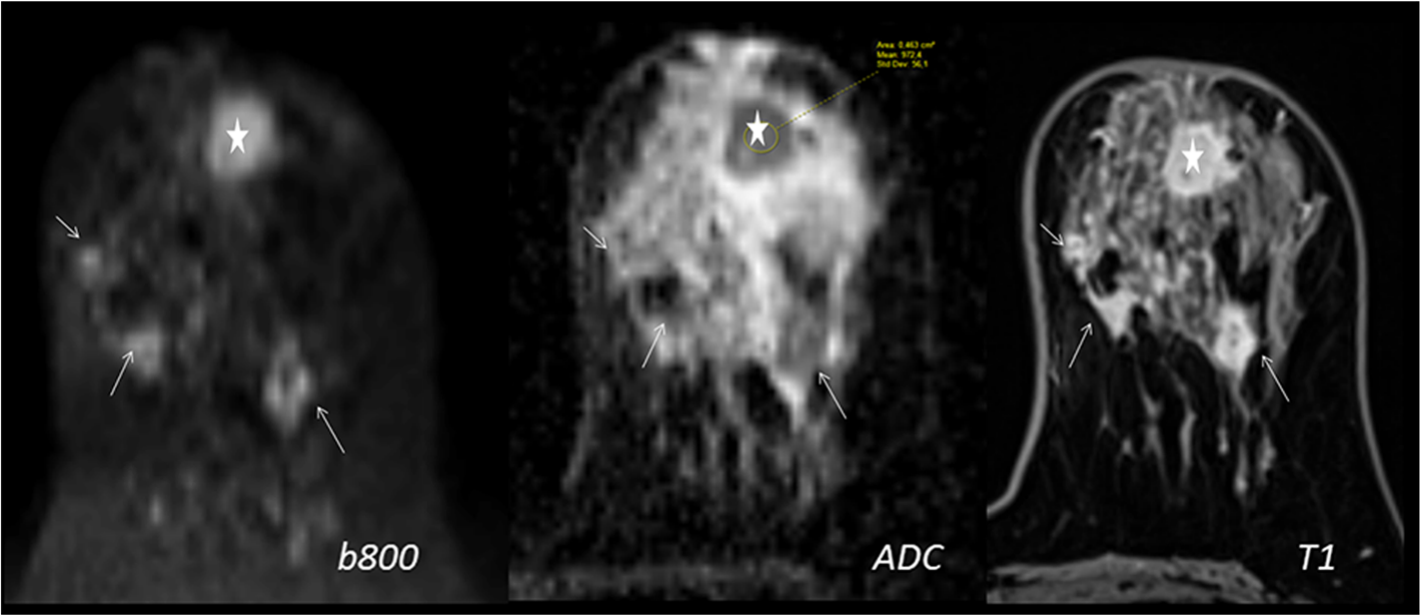
A multicentric breast cancer. Satellite lesions (arrows) could be seen by both DW-MRI and ADC map. They have similar features to the primary lesion (star)

The correct staging and surgical preparation require accurate tumor sizing. One study compared the longest dimension measured on STIR, DW-MRI, ADC map, and the first post-contrast T1-weighted image to pathology results. ADC map displayed the best correlation; however, the difference between ADC map and the other images is unlikely to be clinically significant. This study concluded that ADC map may be a trustworthy technique of correctly sizing tumors without the use of ionizing radiation or contrast material (Fig. 9) [14].

### Monitoring and predicting treatment response

DW-MRI can show the response before morphologic changes and help to identify residual lesions. After neoadjuvant chemotherapy, ADC value can increase within 2–4 days with pathologic evidence with an increase in tumor extracellular space, pleomorphism, large cells apoptosis, and peritumoral oedema and a decrease in cellularity. Therefore, ADC values increase earlier than lesion size decreases (an earlier biomarker for tumor response). That means an early prediction of the effect of chemotherapy, ensuring the best therapeutic effect. DW-MRI can contribute to the evaluation of residual breast cancer following neoadjuvant chemotherapy [4].

### Prognosis

Prognostics of breast carcinoma are histological type and grade, lymph node status and tumor size, molecular biomarkers, and hormone receptors. Although some studies reported some significant correlations between prognostic parameters and ADC values, this point of view is still controversial [15].

### Biopsy guidance

To use DW-MRI for biopsy guidance, the lesion should be a mass larger than 1 cm and can show diffusion restriction [16]. Biopsy guidance by DW-MRI requires some specific equipment, is much more expensive, and is less practical than ultrasonography guidance.

## What are the new developments?

These applications are not yet generalized; especially intravoxel incoherent motion (IVIM) and diffusion kurtosis imaging are not commercialized yet for clinical use. These last two requires some additional software.

### Diffusion tensor imaging

**Fig. 10:**
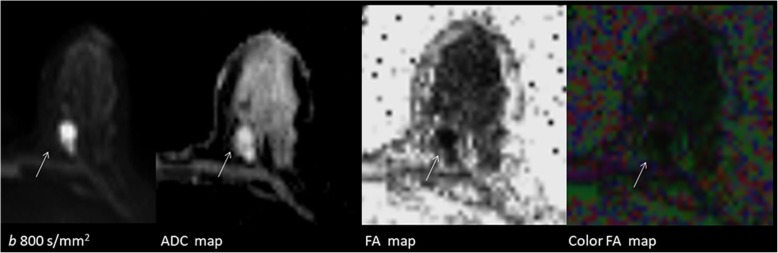
Diffusion tensor imaging maps of a mucinous carcinoma (arrow) of the breast

Diffusion tensor imaging (DTI) is aimed to determine the degree of anisotropic diffusion. For fluids and homogeneous solid materials, diffusion is the same in every direction. This is the isotropic diffusion. However, biological matters are highly organized and typically have altered diffusion coefficients alongside changed directions. This is the anisotropic diffusion. Fractional anisotropy (FA) measures the amount of diffusion asymmetry (Fig. 10). It has been reported that ADC and FA values were statistically different between benign and malignant breast lesions and were significantly correlated to tissue cellularity [17].

### Intravoxel incoherent motion

**Fig. 11:**
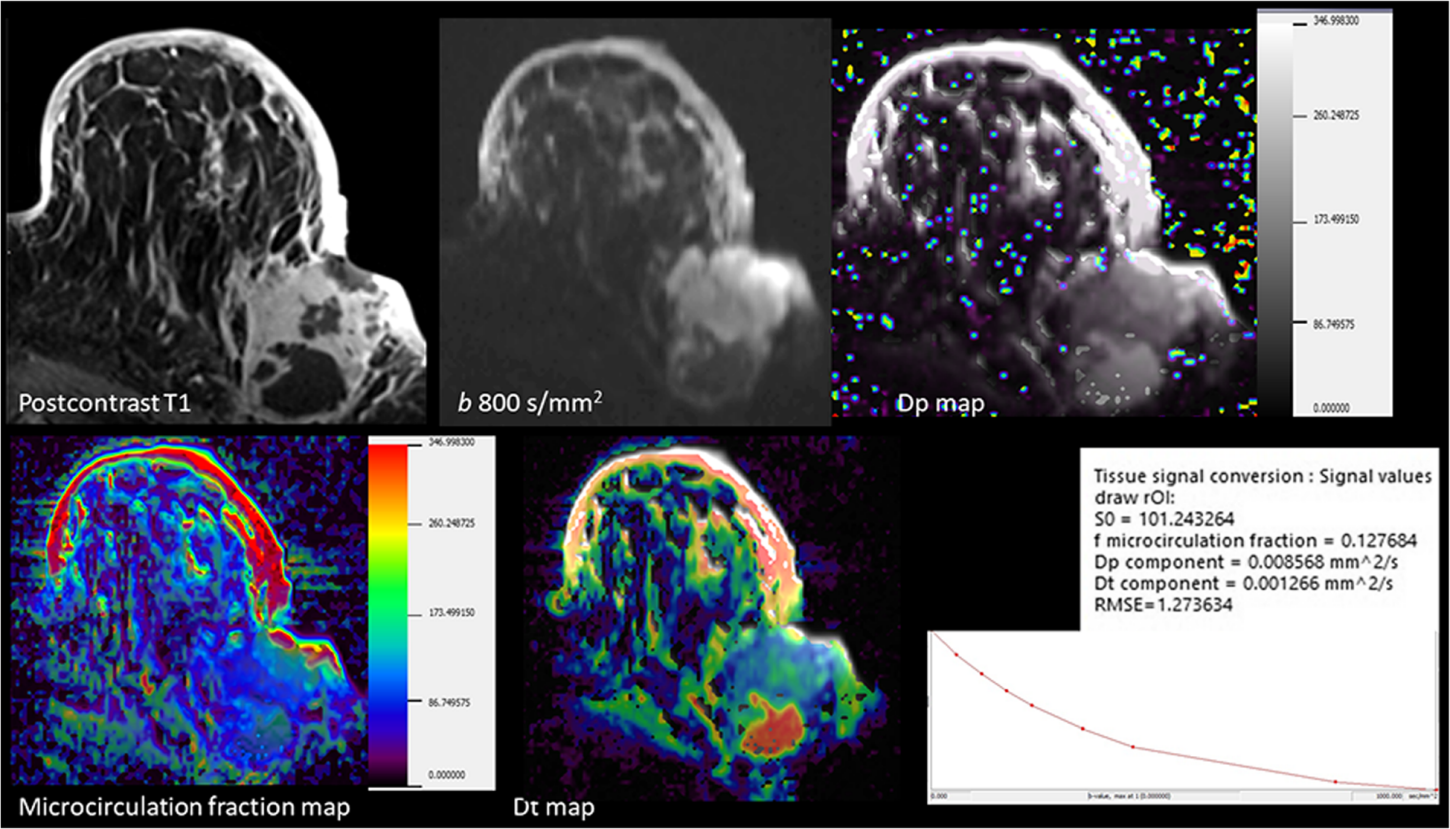
IVIM study of an axillary located breast carcinoma. DW-MRI, postcontrast T1, and corresponding IVIM-derived parameter maps of blood velocity/vessel architecture (pseudodiffusivity-Dp or D*), blood volume (perfusion volume fraction-fp or f), and tissue diffusivity (Dt). The signal decay of breast lesion at nine *b* values of 0, 50, 100, 150, 200, 250, 300, 800, and 1400 s/mm^2^

Intravoxel incoherent motion (IVIM) uses the bi-exponential decay model and tries to separate aids of microvasculature and the adjacent parenchyma. The IVIM provides the parameters of cellularity (tissue diffusivity-Dt), blood volume (perfusion volume fraction-fp or f), and blood velocity/vessel architecture (pseudodiffusivity-Dp or D*). Studies discovered a considerable vascular influence in both DCIS and invasive breast lesions (Fig. 11) [18].

### Diffusion kurtosis imaging

**Fig. 12:**
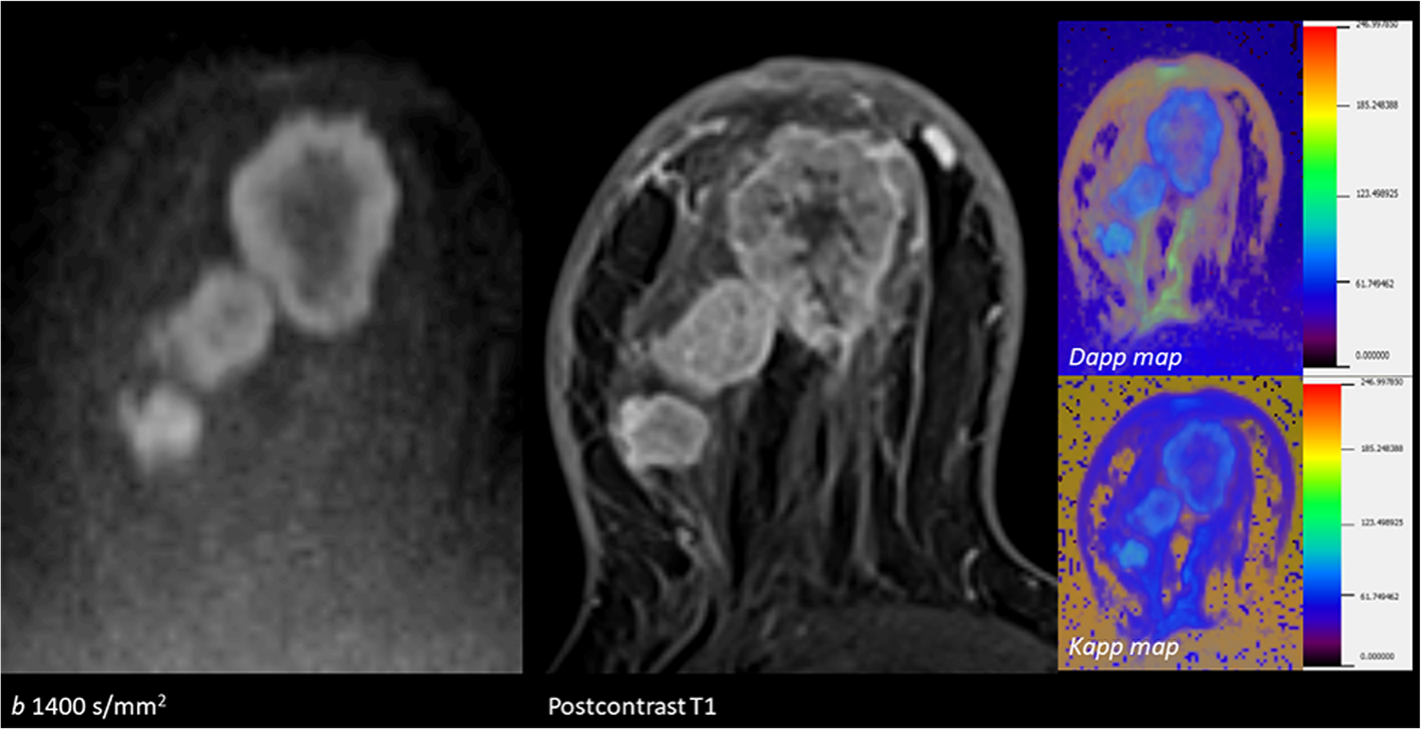
Diffusion kurtosis imaging analysis of multicentric invasive breast carcinoma. DW-MRI, postcontrast T1, and corresponding D apparent (Dapp) and K apparent (Kapp) maps

Standard DW-MRI approach evaluates normal (Gaussian) distribution, which is more accurate for pure liquids or gels. This is most certainly incorrect for complex biologic tissues. Non-Gaussian behavior, specific for intracellular compartment, becomes more noticeable with higher *b* values and longer echo times. It is claimed that diffusion kurtosis imaging better reflects microstructural heterogeneity and tissue complexity and provides accurate characterization and differentiation of breast lesions (Fig. 12) [19]. The value of *D* can be considered as the ADC corrected for kurtosis; *K*, metric for quantifying the shape of a probability diffusion or mean kurtosis averaged over three cardinal directions.

In conclusion, DW-MRI is highly sensitive to changes in the microscopic cellular environment without the need for contrast material injection. It has a short acquisition time and easy processing. It can aid delineation of the extent of disease, monitoring and predicting treatment response, estimating prognosis, and biopsy guidance in suitable circumstances.

## Abbreviations

ADC: Apparent diffusion coefficient
D: ADC corrected for kurtosis
DCE-MRI: Dynamic contrast-enhanced magnetic resonance imaging
Dp or D*: Pseudodiffusivity
Dt: Tissue diffusivity
DTI: Diffusion tensor imaging
DW-MRI: Diffusion-weighted magnetic resonance imaging
FA: Fractional anisotropy
fp or f: Perfusion volume fraction
IVIM: Intravoxel incoherent motion
K: Kurtosis
MRI: Magnetic resonance imaging
nADC: Normalized ADC
NWADCr: Necrosis/wall ADC ratio
SI: Signal intensity
